# Increasing Explicit Sequence Knowledge by Odor Cueing during Sleep in Men but not Women

**DOI:** 10.3389/fnbeh.2016.00074

**Published:** 2016-04-12

**Authors:** Susanne Diekelmann, Jan Born, Björn Rasch

**Affiliations:** ^1^Institute of Medical Psychology and Behavioral Neurobiology, University of TübingenTübingen, Germany; ^2^Center for Integrative Neuroscience (CIN), University of TübingenTübingen, Germany; ^3^Division of Cognitive Biopsychology and Methods, Department of Psychology, University of FribourgFribourg, Switzerland; ^4^Zurich Center for Interdisciplinary Sleep Research (ZiS), University of ZurichZurich, Switzerland

**Keywords:** serial reaction time task, consolidation, slow wave sleep, reactivation, reorganization

## Abstract

Sleep consolidates newly acquired memories. Beyond stabilizing memories, sleep is thought to reorganize memory representations such that invariant structures, statistical regularities and even new explicit knowledge are extracted. Whereas increasing evidence suggests that the stabilization of memories during sleep can be facilitated by cueing with learning-associated stimuli, the effect of cueing on memory reorganization is less well understood. Here we asked whether olfactory cueing during sleep enhances the generation of explicit knowledge about an implicitly learned procedural memory task. Subjects were trained on a serial reaction time task (SRTT) containing a hidden 12-element sequence in the presence of an odor. During subsequent sleep, half of the subjects were re-exposed to the odor during periods of slow wave sleep (SWS), while the other half received odorless vehicle. In the next morning, subjects were tested on their explicit knowledge about the underlying sequence in a free recall test and a generation task. Although odor cueing did not significantly affect overall explicit knowledge, differential effects were evident when analyzing male and female subjects separately. Explicit sequence knowledge, both in free recall and the generation task, was enhanced by odor cueing in men, whereas women showed no cueing effect. Procedural skill in the SRTT was not affected by cueing, neither in men nor in women. These findings suggest that olfactory memory reactivation can increase explicit knowledge about implicitly learned information, but only in men. Hormonal differences due to menstrual cycle phase and/or hormonal contraceptives might explain the lacking effect in women.

## Introduction

There is growing evidence that the processing of memory during sleep fosters the unique ability to detect regular patterns of information in the world and to abstract generalized rules. The explicit knowledge about such regularities is what essentially enables us to adapt to an ever-changing environment. In human studies, sleep supported processes such as pattern detection, abstraction, generalization and the development of explicit knowledge about regularities in materials learnt before sleep (Lewis and Durrant, 2011; Stickgold and Walker, 2013; Landmann et al., 2014). In an assumed process of system consolidation, sleep restructures and redistributes newly encoded memory traces (Walker and Stickgold, 2010; Lewis and Durrant, 2011; Genzel et al., 2014b), which can qualitatively change memory representations and lead to the generation of new knowledge (Payne, 2011a,b). For example, some evidence suggests that sleep following initial learning can make subjects more likely to gain insight into hidden rules (Wagner et al., 2004), integrate distant relations between single elements (Ellenbogen et al., 2007), and abstract schema-like information from learned material (Durrant et al., 2011). Furthermore, sleep can promote the conversion of implicitly learned regular patterns into explicit knowledge about those regularities, which was found to be specifically associated with the amount of slow wave sleep (SWS; Wilhelm et al., 2013).

It is assumed that processes of restructuring and extraction of explicit knowledge originate from the repeated and spontaneous reactivation of memory traces occurring during sleep, and preferentially during Non-rapid eye movement (NREM) sleep (Diekelmann and Born, 2010; Rasch and Born, 2013). A “replay” of learning-associated neuronal activity patterns during sleep after learning was first observed in rats (Wilson and McNaughton, 1994; Nádasdy et al., 1999) and is likewise found in humans with neuroimaging techniques like positron emission tomography (Peigneux et al., 2004). Reactivation does not only occur spontaneously during sleep but can also be triggered by learning-associated cues such as odors and sounds (Oudiette and Paller, 2013). In a visuo-spatial object location task, re-exposing subjects to a learning-associated odor during sleep, specifically during SWS, distinctly enhances memory recall (Rasch et al., 2007), stabilizes memories against subsequent interference (Diekelmann et al., 2011) and accelerates endogenous consolidation processes (Diekelmann et al., 2012). Moreover, presenting learning-associated cues during sleep was found to bias neuronal replay toward the memory that was associated with the respective cues (Bendor and Wilson, 2012).

Reactivation cues presented during sleep also proved effective in enhancing implicit procedural memory of tapping a specific sequence of key presses (Antony et al., 2012; Schönauer et al., 2014). In these studies, auditory tones were paired with different elements of the trained sequence and were subsequently presented again during sleep. Adopting this approach, a recent study reported that cueing with sequence-associated tones during sleep likewise enhances the extraction of explicit knowledge about the underlying sequence, which was specifically associated with sleep spindles (Cousins et al., 2014). Whether this benefit of cueing for explicit knowledge in procedural memory is specific for cueing with auditory tone sequences or whether similar cueing effects can be obtained with a simple contextual odor stimulus is unknown. The only previous study that used odor cues to reactivate procedural memory during sleep did not observe an effect on the performance of finger sequence tapping, however, this study did not test for explicit sequence knowledge (Rasch et al., 2007).

Here we tested the extraction of explicit sequence knowledge by presenting procedural learning-associated odor cues during post-training sleep. Subjects were trained on an implicit serial reaction time task (SRTT), which followed a hidden 12-element sequence, in the presence of a distinct odor. During subsequent periods of SWS subjects were either re-exposed to the odor or received an odorless vehicle. In the next morning, the subjects were asked to explicitly generate the underlying sequence. We hypothesized that odor-induced memory reactivation during SWS enhances explicit sequence knowledge. Since previous findings also suggest that sleep affects memory consolidation processes differently in men and women (Genzel et al., 2012, 2014a), we additionally explored possible sex differences in the odor reactivation effect.

## Materials and Methods

### Participants

A total of 40 healthy right-handed volunteers participated in the experiment. Data from four subjects were excluded due to poor sleep quality, being defined as meeting one or more of the following criteria: (1) more than 90 min sleep latency; (2) more than 140 min awake after sleep onset; (3) less than 20 min SWS. The remaining 36 subjects were aged between 18 and 35 years (mean ± SD: 21.9 ± 3.6 years; 19 females). Of the female subjects, four reported to take hormonal contraceptives, four were in the follicular phase (days 1–14) and 10 in the luteal phase (days 15–28) of their menstrual cycle at the time of the experiment (this information was missing for one woman). Participants were randomly assigned to the “odor” group (*n* = 18; 11 females) or the “vehicle” group (*n* = 18; 8 females). None of the participants had a history of any neurologic, psychiatric or internal medical disease. They reported to have a normal sense of smelling and did not have any nasal infections on the day of the experiments. They also did not report any sleep disturbances during the 4 weeks prior to the experiments and were used to sleep for 7–8 h per night according to a regular sleep schedule (habitual sleep time ~23:00–07:00 h). Subjects were not allowed to ingest any caffeine or alcohol containing drinks or to take naps during the experimental day. All participants were accustomed to sleeping under laboratory conditions by spending an adaptation night in the sleep laboratory including placement of the electrodes and wearing the nasal mask for odor delivery, with at least one night of sleep at home between the adaptation night and the experimental night. The experiments were approved by the local ethics committee of the University of Lübeck and all subjects gave written informed consent in accordance with the Declaration of Helsinki.

### Serial Reaction Time Task (SRTT)

**Figure 1:**
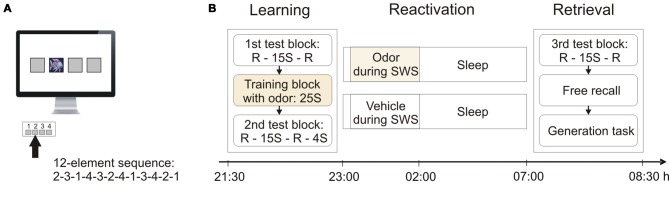
**Serial reaction time task (SRTT) and experimental procedures. (A)** Participants were instructed to press one of four buttons whenever a visual cue (picture of a fish) appeared in one of four corresponding squares on the screen. Unbeknownst to the participants, the appearance of the cue followed a repeating 12-element sequence. **(B)** During learning, participants performed on the SRTT in a 1st test block with 15 sequence (S) repetitions flanked by random (R) trials, followed by the training block containing 25 sequence repetitions with concurrent odor presentation, and the 2nd test block with 15 sequence repetitions flanked by random trials and four final sequence repetitions. During the subsequent 8-h sleep period, half of the subjects were again presented with the odor during slow wave sleep (SWS), whereas the other half received an odor-less vehicle. At retrieval testing in the morning, participants performed on a 3rd test block of the SRTT and were then tested on their explicit knowledge about the sequence in a free recall test and a generation task.

We used a version of the SRTT that was modified after Brown and Robertson (2007b). Four gray squares (positions 1–4) were centrally arranged on a computer screen (Figure 1A). A distinct visual cue (the image of a fish) could appear at any of these four positions. The four positions corresponded to four designated keys on the keyboard. The participants were instructed to press the corresponding key as fast and accurate as possible whenever the cue appeared at one of the four positions. They were required to leave the four fingers (except for the thumb) of their non-dominant left hand on the respective keys. Upon a response, the cue disappeared and reappeared on the next position after an inter-stimulus-interval of 400 ms. Only correct key presses were considered for analysis. Unbeknownst to the participants, the appearance of the cue followed a repeating deterministic 12-element sequence (2-3-1-4-3-2-4-1-3-4-2-1).

Initial learning was composed of three blocks (Figure 1B): The first “test block” started with 48 random trials followed by 15 repetitions of the sequence (i.e., 180 trials) and another 48 random trials. The following “training block” consisted of 25 repetitions of the sequence (i.e., 300 trials). During this training block, all subjects were presented with the experimental odor via a nasal mask (see below). Odor presentation followed an alternating pattern of 5 key presses on-/5 key presses off-phases to reduce habituation. Following the training block, subjects completed the second “test block” which entailed 48 random trials, followed by 15 repetitions of the sequence (i.e., 180 trials), another 48 random trials, and finally four more repetitions of the sequence (i.e., 48 trials). No odor was presented during the two test blocks. Learning performance, i.e., the skill at the end of learning, was calculated as the difference between mean reaction time during the last four sequences of the 15 sequence repetitions in the second test block (i.e., trials 133–180) and the mean reaction time during the ensuing 48 random trials of the second test block.

At retrieval testing, subjects completed the third “test block”, which was identical to the first test block, i.e., consisting of 48 random trials, 15 repetitions of the sequence (i.e., 180 trials), and another 48 random trials. Retrieval performance, i.e., the skill at retrieval, was calculated as the difference between the mean reaction time during the last four sequences of the 15 sequence repetitions in the third test block (i.e., trials 133–180) and mean reaction time during the ensuing 48 random trials of the third test block. Following the third test block, subjects were tested on their explicit knowledge about the sequence (Figure 1B). At this point, all subjects were informed that there was an underlying sequence of cue appearances in the task. Subjects were then asked to try to recall this sequence. In a free recall test they were presented with a black computer screen and were asked to tap the sequence freely using the keys that had been used for the SRTT. All subjects were allowed 48 key presses. In accordance with previous studies (Cousins et al., 2014), the analyses focused on the first 12 key presses, such that subjects had the opportunity to tap the entire sequence once. The number of correctly recalled sequence elements was used as a measure of free recall (ranging from 0 to 12). An individual sequence element was only counted as correct if it was included within a correctly recalled sequence segment of at least three consecutive elements (Willingham and Goedert-Eschmann, 1999). [Note that analyses including all 48 key presses did not reveal any significant effects (all *p* > 0.20)]. Finally, participants completed a generation task. In this task, subjects were presented with two succeeding elements of the sequence and were asked to indicate at which position the cue would appear next, i.e., they had to complete a triplet. All possible triplets had to be completed twice (24 triplets altogether) and the percentage of correctly predicted positions was used as a measure of sequence generation. Odor was never presented during any of the parts of the retrieval session.

### Design and Experimental Protocol

**Figure 2:**
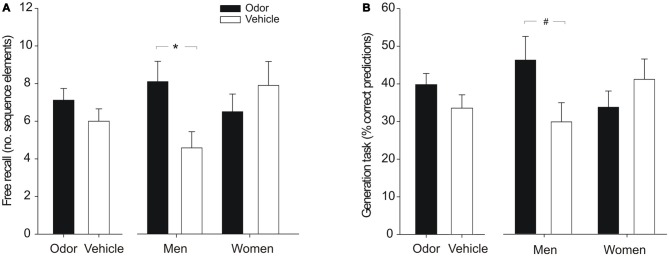
**Explicit sequence knowledge after odor cueing during sleep. (A)** Free recall of the sequence did not differ significantly between the odor and vehicle groups in the whole sample (left panel). Analyzing male and female participants separately showed that odor cueing during SWS increased free recall of the sequence in men but not in women (right panel). Free recall is displayed as the number of correctly recalled sequence elements (range between 0–12). **(B)** Whereas performance on the generation task was not significantly increased by odor stimulation in the whole sample (left panel), odor reactivation tended to improve the ability to generate the sequence in men but not in women (right panel). Generation task performance refers to percent correct predictions of the next cue position upon presentation of two successive elements of the sequence. Mean ± SEM are shown. *p < 0.05, ^#^*p* < 0.08.

Subjects reported to the laboratory at 20:30 h and were prepared for the experiment by attaching the electrodes for sleep recordings and a nasal mask for odor delivery. Next, subjects filled out a mood questionnaire and performed on a vigilance task and an odor detection test (to ensure normal olfactory sensitivity). Learning the SRTT started at 21:30 h. After learning, the odor detection test was repeated. Subjects again filled out the mood questionnaire and performed the vigilance task. They went to bed at 23:00 h and were allowed to sleep normally for 8 h. The olfactory stimuli were presented during SWS in the first 3 h after sleep onset. Half of the subjects received the odor, whereas the other half was presented with an odorless vehicle (Figure 2B). Presentation of the olfactory stimulation started as soon as online polysomnographic recordings indicated more than 20% delta waves (i.e., the presence of SWS) during a 30 s period. The stimulation was interrupted whenever polysomnographic signs of arousal, awakening, or changes in sleep stage appeared. The participants as well as the experimenter were entirely unaware whether odor or vehicle was applied on a given night. In each experimental night, the olfactometer contained odor and vehicle, and the selection was performed automatically by a pre-programmed algorithm unknown to the experimenter. Stimulation during sleep followed an alternating pattern of 30 s on-/30 s off-phases to reduce habituation. Subjects were awakened at 7:00 h in the next morning. The electrodes and the nasal mask were removed and participants had the opportunity to take a shower. Next, subjects again filled out the mood questionnaire and performed the vigilance task. Retrieval of the SRTT started at 8:00 h. Finally, subjects completed the mood questionnaire and the vigilance task one last time, and were asked whether they had smelled the odor during sleep. Subjects in the odor group and the vehicle group did not differ in whether they believed to have smelled the odor (*χ^2^*(2) = 1.85, *p* > 0.35; odor group, “yes”: *n* = 3, “no”: *n* = 11, “don’t know”: *n* = 4; vehicle group, “yes”: *n* = 4, “no”: *n* = 7, “don’t know”: *n* = 7).

### Odor Delivery and Substance

The applied odor was isobutyraldehyde (IBA; Sigma-Aldrich, Germany; similarly used in Diekelmann et al., 2011; Rihm et al., 2014) diluted in odorless mineral oil (1, 2-propanediol; Sigma-Aldrich, Germany) at a concentration of 1:200. The odorless mineral oil served as stimulus in the vehicle control condition. The experimental odor was delivered via a 12-channel computer-controlled olfactometer. The olfactometer was placed in a separate room (adjacent to the subject’s room) and was connected to the subject’s mask via teflon tubes, which allowed regulating the odor stimulation without disturbing the subject.

### Control Tasks

Vigilance was assessed in a standardized test that required pressing as fast as possible a button whenever a big red disc appeared on a computer screen. On 40 trials the subjects had to fixate their gaze on a centrally located cross, displayed for 500–1000 ms on a white screen. Then, in 35 trials, a red disc appeared and in five random no-go trails, the screen remained white. Reaction times averaged across the 35 go trials served as a measure of vigilance. Subjective mood was assessed using the short form of the German version of the Multidimensional Mood Questionnaire (Steyer et al., 1994). The subjects indicated on five-point Likert scales how well 12 different adjectives described their current feeling. The adjectives were then combined into three different bipolar dimensions: “good mood—bad mood”, “alertness—tiredness”, and “calmness—restlessness”, with high values indicating the positive pole and low values indicating the negative pole of each dimension (range of values between 4 and 20). The odor detection test required subjects to indicate the presence or absence of the experimental odor stimulus on 10 trials. All participants completed the vigilance task and the mood questionnaire four times: before learning, after learning, before retrieval, and after retrieval. The odor detection test was only completed before learning and after learning, since no odor was presented at retrieval. For all control tasks the values before and after learning as well as the values before and after retrieval were averaged.

### Sleep Recordings

Polysomnography included electroencephalographic (EEG), electromyographic (EMG) and electrooculographic (EOG) recordings. For EEG recordings six electrodes were placed on the scalp (at positions F3, F4, C3, C4, P3, P4 according to the international 10–20 system), with a reference electrode on the nose. EMG and EOG recordings were obtained from two electrodes placed on the chin and above and below the eyes, respectively. Polysomnographic recordings were visually scored offline by two experienced scorers according to standard criteria as wake, sleep stages 1, 2, 3, 4 and rapid eye movement (REM) sleep (Rechtschaffen and Kales, 1968). Additionally, sleep was visually inspected online by the experimenter to identify SWS for odor stimulation.

Sleep spindles were identified in NREM sleep (i.e., sleep stage 2, 3 and 4). Spindles were detected automatically using a custom-made Software tool (SpindleToolbox, version 1.1) that was based on an algorithm adopted from previous studies (Mölle et al., 2002; Wilhelm et al., 2011). Briefly, first the power spectrum of each subject was calculated, enabling the user to visually detect the peak of the sigma frequency band in each individual. Then, the root mean square (rms) of the bandpass-filtered signal in the range of ± 1.5 Hz around the detected spindle peak of each 200 ms interval was calculated, and the events in which the rms signal exceeded a constant threshold of 5 μV for 0.5–3 s were counted as spindles. The mean peak spindle frequency, spindle count (total number of spindles), spindle density (number of spindles in 30 s), mean spindle length, and mean spindle peak-to-peak amplitude were analyzed.

### Statistical Analyses

Statistical testing for the free recall test and the generation task was initially performed with unpaired t-tests to compare odor and vehicle groups. Follow-up analysis of variance (ANOVA) tested for possible sex differences with the between-subjects factors “odor/vehicle” and “men/women”. Because the subgroups of men and women differed in the amount of SWS and REM sleep, respectively (see below), time spent in SWS and REM sleep was introduced as covariates in the ANOVA as well as in the respective *post hoc* tests (*p*-values without covariates are provided in brackets). *Post hoc* tests are reported uncorrected. For the analysis of free recall, three subjects had to be excluded because of wrong button presses, i.e., these subjects pressed buttons other than the four dedicated sequence buttons (one man from the odor group, one man from the placebo group, and one woman from the placebo group). The skill in the SRTT was first analyzed with an overall ANOVA with the within-subjects factor “learning/retrieval” and the between-subjects factor “odor/vehicle”, and a follow-up ANOVA then included the additional between-subjects factor “men/women” and the covariates SWS and REM sleep. Sleep parameters were analyzed using ANOVAs with the between-subjects factors “odor/vehicle” and “men/women”; analyses of the control tasks included the additional within-subjects factor “learning/retrieval”. *Post hoc* comparisons relied on paired and unpaired t-tests and are reported uncorrected. Greenhouse-Geisser correction of degrees of freedom was applied when appropriate. Correlations were calculated using Pearson’s product moment correlation and were Bonferroni-corrected for the number of tests analyzed. A value of *p* < 0.05 was considered significant.

## Results

### Explicit Sequence Knowledge

Free recall of the underlying SRTT sequence was numerically enhanced in subjects who had received the odor stimulation during sleep compared to the vehicle group, but this difference failed to reach significance (odor vs. vehicle: 7.12 ± 0.62 vs. 6.00 ± 0.66 correctly recalled sequence elements, *t*_(31)_ = 1.23, *p* = 0.23; Figure 2A). Analyzing male and female subjects separately, showed that odor reactivation during sleep had different effects on free recall performance in men and women. Odor stimulation significantly increased the number of correctly recalled elements of the sequence in men [odor vs. vehicle: 8.11 ± 1.08 vs. 4.59 ± 0.86; *F*_(1,11)_ = 5.81, *p* = 0.035 (without covariates: *p* = 0.044)], whereas in women no such difference was observed (odor vs. vehicle: 6.51 ± 0.94 vs. 7.91 ± 1.27, *F*_(1,14)_ = 0.60, *p* = 0.45; interaction “odor/vehicle” × “men/women”: *F*_(1,27)_ = 5.78, *p* = 0.023 (without covariates: *p* = 0.068); main effects “odor/vehicle” and “men/women”: both *F*_(1,27)_ < 1.70, *p* > 0.20; Figure 2A). A similar pattern of results was evident for performance in the generation task. Considering the whole sample, odor participants were numerically but non-significantly superior to the vehicle group in the percentage of correct sequence predictions (odor vs. vehicle: 39.81 ± 2.96% vs. 33.56 ± 3.54%; *t*_(34)_ = 1.35, *p* = 0.18; Figure 2B). When analyzing men and women separately, odor reactivation tended to increase the prediction performance in men [odor vs. vehicle: 46.2 ± 6.3% vs. 29.8 ± 5.1%; *F*_(1,13)_ = 3.63, *p* = 0.079 (without covariates: *p* = 0.019)] but not in women (odor vs. vehicle: 33.7 ± 4.3% vs. 41.1 ± 5.4; *F*_(1,15)_ = 0.89, *p* = 0.36; interaction “odor/vehicle” × “men/women”: *F*_(1,30)_ = 4.91, *p* = 0.035 (without covariates: *p* = 0.011), main effects “odor/vehicle” and “men/women”: both *F*_(1,30)_ < 1.90, *p* > 0.18; Figure 2B).

To explore whether the lacking effect of odor reactivation on explicit sequence knowledge in female subjects might be related to differences in sex hormone concentrations at different time points of the menstrual cycle or due to the use of hormonal contraceptives, we compared the performance of women taking oral contraceptives (*n* = 4) with those in the follicular phase (*n* = 4) and in the luteal phase of their menstrual cycle (*n* = 10). Explicit sequence knowledge did not differ between women in the different hormonal states, neither in free recall (contraceptives vs. follicular vs. luteal: 7.00 ± 1.61 vs. 6.50 ± 1.39 vs. 7.20 ± 0.88; *F*_(2,14)_ = 0.09, *p* = 0.91) nor in the generation task (41.70 ± 5.70% vs. 35.40 ± 3.60% vs. 38.50 ± 5.70%; *F*_(2,15)_ = 0.46, *p* = 0.64). Follow-up analyses on the effects of odor cueing in the different hormonal states were not possible due to the low number of women in the different subgroups (odor group: contraceptives *n* = 1, follicular *n* = 1, luteal *n* = 9; placebo group: contraceptives *n* = 3, follicular *n* = 3, luteal *n* = 1).

### Serial Reaction Time Task Performance

**Figure 3:**
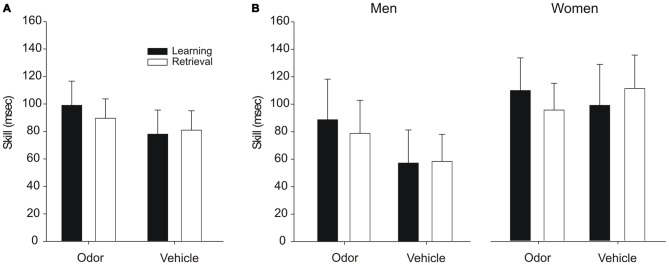
**Performance on the SRTT after odor reactivation during sleep.** Odor presentation during SWS did not affect the skill level in the SRTT, neither in the whole sample **(A)** nor in men and women separately **(B)**. The skill level is presented as the difference between the last random trials and the preceding last sequence trials (in ms) at the end of learning (2nd test block) and at retrieval (3rd test block), respectively. Mean ± SEM are shown.

Performance on the SRTT was not affected by odor reactivation during sleep. Participants in the odor group and the vehicle group expressed comparable skill levels when analyzing the whole sample (*F*_(1,34)_ < 0.70, *p* > 0.40, for main effects “odor/vehicle” and “learning/retrieval” and interaction “odor/vehicle” × “learning/retrieval”; Figure 3A) as well as when analyzing men and women separately (*F*_(1,30)_ < 1.24, *p* > 0.27, for main effects “odor/vehicle” and “learning/retrieval” as well as for all possible interaction effects; Figure 3B). Overall, women tended to perform better than men (*F*_(1,30)_ = 3.26, *p* = 0.081, for main effect “men/women”). Importantly, participants in all groups had acquired a skill by the end of learning as evidenced by significantly faster reaction times during the sequence trials at the end of learning compared to the subsequent random trials (all *p* < 0.05). Moreover, this skill level did not change over the night from learning to retrieval in any of the groups (all *p* > 0.35).

### Sleep Data and Control Tasks

Participants in all groups showed normal nocturnal sleep patterns (Table 1). Overall, groups were comparable in sleep patterns with regard to sex (all main effects “men/women”: *p* > 0.15) as well as with regard to odor cueing (all main effects “odor/vehicle”: *p* > 0.13; except for total sleep time: *p* = 0.02, but *post hoc* comparisons: all *p* > 0.10). A few differences in sleep stage distribution emerged on the level of subgroups of odor and vehicle in men and women (*p* < 0.05 for interaction “odor/vehicle” × “men/women” for Wake, stage 4, SWS, and REM sleep; all other *p* > 0.14). *Post hoc* comparisons revealed that men displayed less SWS in the odor group compared to the vehicle group (*p* = 0.047), women displayed more REM sleep in the odor group compared to the vehicle group (*p* = 0.008), and both men and women differed marginally in stage 4, with men showing less stage 4 and women showing more stage 4 in the odor group (both *p* < 0.09; all other *p* > 0.10). Importantly, including the amount of SWS and REM sleep as covariates in the analyses of explicit sequence knowledge did not change any of the results (see above). Moreover, there were no significant correlations between the time spent in any of the sleep stages and explicit sequence knowledge (i.e., free recall and generation task performance), neither in the overall sample nor in the different subgroups of subjects. Spindle measures did not differ between men and women in the odor and vehicle groups. Women tended to display a higher peak spindle frequency than men (13.62 ± 0.12 vs. 13.31 ± 0.13 Hz, *p* = 0.086 for main effect “men/women”), but this difference was not affected by odor reactivation (*p* > 0.35 for main effect “odor/vehicle” and interaction; *p* > 0.13 for all other comparisons). The number of cueing events during SWS was comparable between odor and vehicle groups in men and women (all *p* > 0.14, see Table 1). Neither any of the spindle measures nor the number of cueing correlated significantly with explicit sequence knowledge.

**Table 1:** **Sleep data**.

	Men		Women	
	Odor	Vehicle	Odor	Vehicle
**Sleep stage distribution**
TST	471.6 ± 6.3	456.6 ± 5.7	471.1 ± 2.7	453.8 ± 11.0
Wake	34.3 ± 11.5	19.6 ± 3.5	18.8 ± 3.1	40.0 ± 14.3
S1	11.6 ± 2.9	10.9 ± 2.6	14.2 ± 2.4	13.6 ± 4.3
S2	279.0 ± 14.5	249.2 ± 12.4	257.3 ± 8.7	266.2 ± 17.3
S3	41.9 ± 4.9	49.1 ± 4.4	47.1 ± 3.1	43.4 ± 3.9
S4	16.4 ± 6.1	32.7 ± 5.4^#^	38.4 ± 5.7	22.6 ± 6.3^#^
SWS	58.4 ± 9.2	81.7 ± 6.4*	85.5 ± 5.6	65.9 ± 8.7^#^
REM	86.6 ± 8.7	93.0 ± 5.8	93.5 ± 5.2	66.8 ± 7.5*
SL	30.4 ± 4.4	30.8 ± 5.2	20.4 ± 2.6	29.8 ± 7.0
**Spindle measures**
Peak spindle frequency	13.2 ± 0.2	13.4 ± 0.2	13.5 ± 0.2	13.7 ± 0.2
Spindle count	1670.3 ± 87.3	1702.8 ± 73.1	1711.9 ± 69.7	1612.5 ± 81.7
Spindle density	2.5 ± 0.1	2.6 ± 0.1	2.5 ± 0.1	2.4 ± 0.1
Spindle length	0.88 ± 0.02	0.86 ± 0.02	0.88 ± 0.02	0.85 ± 0.02
Spindle P-P amplitude	29.9 ± 3.0	31.3 ± 2.5	34.4 ± 2.4	32.7 ± 2.8
**Cueing**	46.1 ± 7.4	56.7 ± 6.3	63.5 ± 4.4	56.0 ± 6.5

Sleep stage distribution is presented in minutes. TST, Total sleep time; Wake, time awake after sleep onset; S1–S4, sleep stages 1–4; SWS, slow wave sleep (i.e., the sum of sleep stages 3 and 4); REM, rapid eye movement sleep; SL, sleep latency. Spindles were analyzed in S2, S3, and S4. Peak spindle frequency in Hz, Spindle count—total number of spindles, Spindle density—number of spindles in 30 s, Spindle length—in seconds, Spindle P-P amplitude—spindle peak-to-peak amplitude in μV. Cueing—mean number of cueing events. Data are presented as mean ± SEM. *p < 0.05, ^#^*p* < 0.09 (compared to odor, within the subgroup of men and women, respectively).

Results of the control tasks are summarized in Table 2. Reaction times in the vigilance task did not differ between the odor and vehicle groups in men and women, yet reaction times were generally faster at retrieval than at learning (*F*_(1,32)_ = 11.50, *p* = 0.002, for main effect “learning/retrieval”; *F*_(1,32)_ < 4.15, *p* > 0.05, for all remaining main effects and interactions). Similarly, subjective alertness was generally higher at retrieval compared to learning; however, there were no differences between the odor and vehicle groups in men and women (*F*_(1,32)_ = 24.60, *p* < 0.001, for main effect “learning/retrieval”; *F*_(1,32)_ < 1.10, *p* > 0.30, for all remaining main effects and interactions). Good mood and calmness did not differ between the odor and vehicle groups in men and women at learning as well as at retrieval (*F*_(1,32)_ < 3.90, *p* > 0.05, for all possible effects). Performance on the odor detection task was close to perfect in all groups (92–97% correct responses; *F*_(1,32)_ < 1.70, *p* > 0.20, for all possible effects).

**Table 2:** **Control tasks**.

		Men		Women	
		Odor	Vehicle	Odor	Vehicle
**Vigilance**
	Learning	274.0 ± 11.8	295.8 ± 9.9	273.0 ± 9.4	279.6 ± 11.0
	Retrieval	257.4 ± 7.7	287.4 ± 6.5	261.0 ± 6.2	269.6 ± 7.2
**Mood**
Good	Learning	16.1 ± 0.7	16.1 ± 0.6	16.6 ± 0.5	16.8 ± 0.6
mood
	Retrieval	16.7 ± 0.8	15.5 ± 0.7	17.0 ± 0.7	16.2 ± 0.8
Alertness	Learning	10.8 ± 1.0	11.4 ± 0.8	10.6 ± 0.8	12.2 ± 0.9
	Retrieval	14.1 ± 1.0	13.6 ± 0.9	14.2 ± 0.8	14.6 ± 1.0
Calmness	Learning	16.5 ± 0.7	15.1 ± 0.6	17.2 ± 0.6	16.1 ± 0.7
	Retrieval	15.9 ± 0.7	15.0 ± 0.6	16.9 ± 0.6	16.4 ± 0.7
**Odor detection**
	Learning	96.7 ± 2.7	93.7 ± 2.3	95.8 ± 2.2	92.5 ± 2.5

*Vigilance refers to reaction times in ms. The mood questionnaire reveals ratings between 4 and 20, with higher numbers indicating better mood, feeling more alert, and being calmer. Odor detection values refer to % correct responses. Mean ± SEM are shown*.

## Discussion

The efficacy of a contextual odor cue to facilitate the conversion from implicit into explicit sequence knowledge in a procedural memory task was hitherto unknown. Here we show that the presentation of learning-associated olfactory reminder cues during SWS can enhance the extraction of explicit knowledge about an implicitly learned SRTT motor sequence. Interestingly, this odor cueing effect was only evident in men but not in women. These results corroborate and extend recent findings by Cousins et al. (2014), showing increased explicit sequence knowledge in a similar SRTT following reactivation with auditory cues during SWS. The study sample of Cousins et al. included male and female participants but sex differences were not reported.

### Explicit Sequence Knowledge

Mounting evidence indicates that targeted memory reactivation by external olfactory or auditory cues during SWS facilitates neuronal reactivation processes (Oudiette and Paller, 2013; Cairney et al., 2014; Rihm et al., 2014). A study in rats showed that such reactivation cues can bias neuronal replay towards activity patterns from the prior learning episode at the cost of the uncued memory content (Bendor and Wilson, 2012). Previous studies applying visuo-spatial learning paradigms found that the re-exposure to learning-associated olfactory or auditory cues during SWS activates hippocampal and cortical areas that are known to be implicated in the learning of visuo-spatial tasks (Rasch et al., 2007; Diekelmann et al., 2011; van Dongen et al., 2012). Apart from a mere strengthening, sleep is well-known to foster memory reorganization assumed to underlie processes such as gaining insight into hidden structures, schema abstraction, and the extraction of gist knowledge (Lewis and Durrant, 2011; Payne, 2011a; Stickgold and Walker, 2013; Landmann et al., 2014), and there is first evidence that auditory cueing during SWS enhances these processes, e.g., increasing grammatical generalization (Batterink and Paller, 2015) and the generation of explicit sequence knowledge (Cousins et al., 2014). Our findings show that a single contextual olfactory cue repeatedly presented during SWS can likewise increase the extraction of explicit sequence knowledge from an implicitly learned procedural memory task. Whether this reorganization also relies on sleep-dependent neuronal replay of learning-related activity patterns has to be scrutinized in future studies using neuroimaging techniques.

### Procedural Skill

Whereas in the present study odor cueing enhanced explicit sequence knowledge in men, overall motor performance, i.e., the skill level in the SRTT, was not affected. This finding is in line with an earlier study that did not observe any beneficial effect of odor cueing during SWS on performance in the procedural finger sequence tapping task, despite a pronounced enhancement of declarative visuo-spatial memories (Rasch et al., 2007). This study also did not find any benefits of odor reactivation during REM sleep or during wakefulness for finger sequence tapping, arguing against the possibility that procedural memory might be more responsive to memory reactivation during REM sleep or wakefulness. Other studies have shown that a reactivation-induced enhancement of procedural memory consolidation during sleep, and specifically during SWS, is indeed possible, but so far this effect has only been revealed with auditory reminder cues (Antony et al., 2012; Cousins et al., 2014; Schönauer et al., 2014).

It can be speculated that odor, as compared with tones, is more of a general context cue that is not specific enough to trigger reactivation of the motor component of procedural memories. Particular tones can be paired with single finger movements of a motor sequence, resulting in a kind of melody that is mapped onto the sequence. Cueing with this melody essentially amounts to exposing subjects to the specific sequence again during sleep. Odor cues, on the other hand, presumably become associated with the entire task and the learning context, being simpler contextual cues that are more unspecific with regard to single key presses and the sequence. Alternatively, auditory and olfactory cues might target different aspects of the procedural memory trace. It is assumed that motor sequence memories include non-declarative aspects (i.e., the implicit motor component) and declarative aspects (i.e., the explicit knowledge component), both of which rely on different brain regions and interact to a certain extent (Schendan et al., 2003; Robertson et al., 2004; Brown and Robertson, 2007b; Albouy et al., 2008). Auditory reactivation cues might target both the non-declarative motor aspects as well as the declarative knowledge aspects of the task, thereby facilitating more efficient motor performance together with better explicit knowledge about the underlying sequence (Cousins et al., 2014). This idea is in line with evidence showing that auditory stimuli during sleep are processed in wide-spread cortical and subcortical areas (Czisch et al., 2009), with the auditory system having particularly prominent connections to motor regions (Zatorre et al., 2007; Boyer et al., 2013). Olfactory cues, on the other hand, might act in a more selective way to trigger reactivation of the declarative knowledge aspects of the task, leaving the non-declarative motor aspects unchanged. Indeed, olfactory processing areas are directly and strongly connected to medio-temporal and hippocampal areas known to be involved in declarative memory (Rasch et al., 2007; Diekelmann et al., 2011), whereas olfactory projections to motor regions are much weaker and less direct (Zelano and Sobel, 2005). Note that the effects of external cueing during sleep might not be directly comparable to the “natural” effects of uncued sleep-dependent memory consolidation. External cueing might not simply trigger or facilitate the natural consolidation process, but might target only certain aspects or bias the consolidation process toward the cued direction (Bendor and Wilson, 2012).

### Sex Differences

We found that male and female subjects responded differently to memory cueing during sleep. While odor cueing enhanced explicit sequence knowledge in men, women showed no cueing effect. Based on the present data, we can only speculate with regard to the reasons of this observed difference. Considering previous evidence (Genzel et al., 2012, 2014a), differences in the hormonal status can be considered a prime candidate as a potential explanation. Sleep-dependent memory consolidation has been shown to differ in men and women and these differences were found to be associated with sex hormones in women. Genzel et al. (2012) reported an improving effect of sleep on memory in men, whereas in women this effect was only evident in the luteal phase of the menstrual cycle but not in the follicular phase. Furthermore, sleep-dependent memory consolidation in women was associated with concentrations of estrogen and progesterone. Another study of this group observed no memory-enhancing effect of sleep in women using oral hormonal contraceptives (Genzel et al., 2014a). Thus, in the present study, differences in sex hormone concentrations during different phases of the menstrual cycle or the use of hormonal contraceptives might have prevented the expression of a cueing effect in women. However, the present study was not designed to test this question directly. The sample size was too small to analyze odor reactivation effects in the different subsets of female participants and the different hormonal states were not evenly distributed across the experimental groups (i.e., odor stimulation vs. vehicle). Also *post hoc* comparisons are reported uncorrected and are only marginally significant when corrected for multiple comparisons. Therefore, any conclusions with regard to a potential odor-induced reactivation effect on explicit sequence knowledge in women as well as a potential dependency on menstrual cycle phase and/or use of oral contraceptives remain tentative. When testing larger subsamples of different hormonal states, it might even turn out that cued reactivation does not fail in all women but only in women in a specific hormonal state, similar to sleep effects for memory consolidation being only observed in a certain hormonal state but not in others states. Future studies need to test for cueing effects during sleep in different hormonal states systematically. Moreover, even when not testing for sex differences, future studies should at least ensure that the hormonal state is counterbalanced across experimental conditions when studying female subjects.

### Caveats

The observed differences between subgroups in sleep stage distribution, and particularly in the amount of SWS, are a major limitation of the present study. Interestingly, these differences do not seem to be related to general sex differences in sleep architecture nor to general effects of odor cueing during sleep. Instead, the differences in sleep parameters seem to emerge at the level of subgroups, suggesting that odor cueing (or odor presentation *per se*) might have different effects on sleep in men and women. It is known that odor perception and discrimination abilities are typically superior in women (Brand and Millot, 2001), with sex differences likewise being evident in neurophysiological responses to odors (Evans et al., 1995; Yousem et al., 1999; Savic et al., 2001). Some studies also suggest that men and women show different patterns of changes in sleep stages when odors such as lavender or peppermint are presented shortly before sleep (Goel et al., 2005; Goel and Lao, 2006). However, we are not aware of any studies on sex differences in sleep architecture upon odor presentation during sleep. Importantly, there are several reasons for why we believe that it is unlikely that the differences in sleep stage distribution affected the main findings of the present study. First, despite the different amount of SWS in the single subgroups, the number of cueing events was comparable, confirming that all subgroups received similar amounts of odor cueing. Second, introducing SWS and REM sleep as covariates into the analyses did not essentially change any of the results. Third, neither the amount of SWS nor any other sleep stage was correlated with any of the memory measures, indicating that the behavioral findings were not directly dependent on the amount of SWS or any other sleep stage. Finally, if a higher amount of SWS and/or cueing resulted in stronger effects for the generation of explicit sequence knowledge, we would have expected stronger effects in the subgroups that obtained more SWS and/or cueing. However, on the contrary, we found that the subgroup with the lowest amount of SWS and the fewest odor stimulations (i.e., the subgroup of men who received the odor) showed the strongest benefit for explicit sequence knowledge. Although these considerations collectively suggest that the present findings were not affected by the observed differences in sleep stages, future studies should replicate these findings and should carefully control for differences in sleep and cueing events.

The differences in sleep patterns might also have been affected by the adaptation night being introduced at least one night before the experimental night. This was done to avoid confounding effects of potential rebound sleep during the experimental night in the case that subjects slept poorly during the adaptation night having taken place directly before the experimental night. It is well known that sleep quality is reduced during the first night of sleep in a new environment, called the “first night effect” (Agnew et al., 1966; Toussaint et al., 1995). Thus, although we can exclude that our results are affected by potential confounds of rebound sleep, we cannot exclude that first night effects might have influenced the results of the present study because of the delay between the adaptation night and the experimental night.

An additional analysis, comparing all women (odor plus vehicle) to the subgroup of men who received vehicle, revealed generally superior performance in explicit sequence knowledge in women. This effect reached significance for free recall (number of correct sequence elements: women 7.06 ± 0.60, men vehicle 4.89 ± 0.82; *t*_(25)_ = 2.10, *p* = 0.046) but not in the generation task (percent correct predictions: women 36.84 ± 2.56%, men vehicle 28.75 ± 5.50%, *t*_(27)_ = 1.53, *p* = 0.14). Importantly, the higher performance level in women is not due to a ceiling effect, considering that the maximum performance (i.e., perfect sequence knowledge) is 12.

Although on a descriptive level, men in the vehicle group seemed to perform generally worse in procedural skill than men in the odor group (Figure 3B), this difference was not significant, neither overall (*p* = 0.11), nor when analyzing the learning session separately (*p* = 0.11). Importantly, this descriptive difference is unlikely to have affected the main findings of the present study, considering that there were no effects of odor cueing on procedural skill but only in explicit sequence knowledge. It could be argued that the procedural skill level influences the generation of explicit sequence knowledge indirectly, such that a higher skill level indicates a better acquisition of the motor sequence, thereby increasing the chance for the odor to increase explicit sequence knowledge. However, if this was true, we would have expected even stronger odor effects in women, who displayed an overall better procedural skill than men, but odor cueing did not affect explicit sequence knowledge in women at all. Finally, procedural skill levels were not correlated with explicit sequence knowledge (all *p* > 0.13). Collectively, these data suggest that the observed findings were not affected by differences in procedural skill level.

Another interesting observation from our data is that there was no general “enhancement” of procedural performance across sleep, i.e., a gain in skill from learning to retrieval (Figure 3). While such an enhancement of performance is consistently found in the widely applied finger sequence tapping task (Walker et al., 2002, 2003), a similar enhancement is not consistently observed in the SRTT (e.g., Brown and Robertson, 2007a; Cousins et al., 2014). For example, Cousins et al. (2014) likewise did not observe a general skill enhancement across sleep in the SRTT in the uncued sequence, despite an increase in explicit sequence knowledge for the cued sequence. Moreover, it has recently been argued that even in the classical finger sequence tapping task, the observed skill enhancement does not reflect an actual sleep-dependent gain in performance but rather depends on certain methodological details of the task (Rickard et al., 2008; Nettersheim et al., 2015), challenging the general concept of sleep-dependent enhancement of procedural memory.

Finally, the present study only tested cueing during SWS, leaving open the question whether cueing during other sleep stages, such as stage 2 sleep or REM sleep, has similar or different effects on the generation of explicit sequence knowledge. Particularly, it has been suggested that REM sleep plays a role in the abstraction and generalization of memories (Stickgold and Walker, 2013), which might also be relevant in the conversion from implicit into explicit knowledge. However, there are only a handful of studies that applied reactivation cues during REM sleep, with these studies revealing mixed results. Some evidence suggests that auditory cueing during REM sleep might affect certain aspects of emotional memory (Hars et al., 1985; Rihm and Rasch, 2015) as well as the ability to discriminate between studied and unstudied items (Sterpenich et al., 2014), while two studies on olfactory cueing during REM sleep found no effects on declarative memory or procedural memory (Rasch et al., 2007; Cordi et al., 2014). Nevertheless, based on the idea that REM sleep fosters memory abstraction and generalization, future studies should apply reactivation cues during REM sleep with the explicit sequence knowledge paradigm to test for this hypothesis directly.

## Author Contributions

SD, JB and BR conceived and designed the study. SD and BR collected the data. SD analyzed the data. SD, JB and BR wrote the article.

## Funding

This work was supported by a grant from the Deutsche Forschungsgemeinschaft (DFG; TR-SFB 654 “Plasticity and Sleep”).

## Conflict of Interest Statement

The authors declare that the research was conducted in the absence of any commercial or financial relationships that could be construed as a potential conflict of interest.
